# Effects of Cadmium, Lead and Mercury on Glial Cells in a Rat Model: A Systematic Review

**DOI:** 10.3390/ijms27198457

**Published:** 2026-09-22

**Authors:** Natalia Gajocha, Danuta Izabela Kosik-Bogacka, Katarzyna Piotrowska

**Affiliations:** 1 Department of Biology, Parasitology and Pharmaceutical Botany, Pomeranian Medical University in Szczecin, Powstańców Wielkopolskich 72, 70-111 Szczecin, Poland; 2 Department of Physiology, Pomeranian Medical University in Szczecin, Powstańców Wielkopolskich 72, 70-111 Szczecin, Poland

**Keywords:** glial cells, cadmium, lead, mercury, rats, heavy metals, neurotoxicity, central nervous system

## Abstract

This review summarizes the effects of heavy metals, including lead (Pb), mercury (Hg), and cadmium (Cd), on glial cells in experimental rat models. The analysis was based on scientific literature from the PubMed database, using keywords related to glial cells, lead, mercury, and cadmium, and rats. The reviewed studies included peer-reviewed original research articles and review papers in English. The reviewed literature highlights the mechanisms underlying heavy metal-induced neurotoxicity and their effects on the central nervous system (CNS). Exposure to Pb, Hg, and Cd was shown to influence glial cell function by promoting oxidative stress, increasing reactive oxygen species production, enhancing inflammatory responses, and disrupting blood–brain barrier integrity. These metals may also affect circadian regulation and alter melatonin distribution within the CNS. These changes contribute to morphological, physiological, and developmental alterations in glial cells. The findings indicate that glial cells represent a valuable model for investigating metal-induced cytotoxicity and neurotoxic mechanisms. Rat strains such as Sprague–Dawley and Wistar are widely used experimental models for studying CNS alterations throughout different stages of development. Understanding the molecular mechanisms of heavy metal toxicity is essential for developing effective strategies to prevent and mitigate the adverse effects of environmental pollutants on the nervous system.

## 1. Introduction

Glial cells are the most abundant and widely distributed non-neuronal cell type in the central nervous system (CNS). They interact closely with neurons, immune cells, and the cerebral vasculature, thereby contributing to the maintenance and surveillance of the physiological microenvironment. In addition to providing structural support, glial cells regulate neuronal metabolism, maintain ionic homeostasis, modulate immune responses, and preserve overall CNS homeostasis. They also represent the first line of defense against physiological disturbances and pathological insults, playing a central role in both CNS homeostasis and disease [1]. Notably, glial cells are among the first parenchymal cells to encounter molecules that penetrate the blood–brain barrier (BBB). This strategic location enables them to exert neuroprotective effects by detecting, sequestering, and limiting the cytotoxic impact of xenobiotics, including heavy metals, thereby helping to protect the CNS from toxic injury [2].

For many years, research on glial cells has been conducted, with numerous studies utilizing laboratory animals. A substantial body of data on glial cell function derives from studies employing rodent glioma cell lines, including polyploid C6, C6, and TR33B [3], as well as rat models. These studies have demonstrated that astrocytes play a critical role in neuroprotection by maintaining ionic balance, removing excess glutamate, and supplying neurons with essential energy substrates, thereby helping to prevent excitotoxicity and oxidative stress [4]. Furthermore, in response to CNS injury, astrocytes and microglia actively participate in the regulation of immune processes by releasing cytokines and modulating inflammation, which supports neuronal protection and promotes tissue regeneration [5]. These mechanisms are particularly important in the context of exposure to toxic factors, including heavy metals, which can disrupt neuronal function and the glial environment.

Humans are continuously exposed to a broad spectrum of potentially hazardous substances present in the environment, including air, water, soil, and food. Among the elements commonly classified as heavy metals, lead (Pb), cadmium (Cd) and mercury (Hg) are recognized as highly toxic environmental contaminants. Their environmental persistence, bioaccumulative potential, and pronounced toxicity make them important public health concerns [6]. In particular, Pb, Cd, and Hg are well-established neurotoxicants capable of adversely affecting both the central and peripheral nervous systems, with exposure associated with a broad range of neurological and neurobehavioral disturbances. Given their potential to disrupt nervous system homeostasis, particular attention has been directed toward their effects on glial cells, which play essential roles in neuronal support, synaptic function, neuroinflammation, and maintenance of the neural microenvironment. Therefore, the aim of this study was to critically analyze the available scientific literature concerning the effects of Pb, Cd, and Hg exposure on the morphology, function, and development of glial cells in rat models. A better understanding of the mechanisms underlying heavy metal-induced glial dysfunction may contribute to the identification of potential neuroprotective strategies and improve our understanding of the adverse effects of these environmental contaminants on the nervous system.

The discovery of Cd dates back to 1817, when Friedrich Stromeyer identified the element in Göttingen, Germany [7]. This metal possesses chalcophilic properties and readily reacts with various chemical compounds including with carbonates. In its +2 oxidation state, Cd forms various cationic and anionic complexes [8]. It is considered one of the most toxic metals. It is used in cells and batteries including nickel–cadmium batteries, alloys, yellow pigments, plastic stabilizers, dyes, and paints, as well as in glass manufacturing and galvanic industry. Cadmium is used in nuclear reactors as a neutron-absorbing material to control the uranium fission reaction through electron capture [9]. Environmental contamination with Cd constitutes a serious threat to human health, as it is regarded as one of the most harmful chemical elements [10]. Although Cd accumulates primarily in the kidneys and liver, Cd poisoning—due to its mechanism of action—can also manifest in other organs and systems, including the CNS. In the CNS, Cd can lead to behavioral disturbances, such as impairments in memory and attention, as well as neurodegenerative diseases like Alzheimer’s or Parkinson’s [11,12,13].

Lead, similarly to Cd, exhibits chalcophilic properties, and its most common mineral form is galena (PbS). Additionally, Pb is a component of many minerals. Lead has been used by humans since antiquity, with evidence of its use dating back to ancient Egypt, China, and the Caucasus. During the Roman period, lead was widely used in the manufacture of pottery and in the construction of aqueducts. By the late Middle Ages, its applications had expanded to include the production of printing type and ammunition [14]. Lead is used in the production of cables, ammunition, battery plates [10], as well as anticorrosive materials and paints. Due to its high toxicity and widespread environmental contamination, Pb poses a significant threat to both human health and the natural environment [15].

The history of Hg use dates back to ancient China, India, and Egypt, where it was primarily employed as a pigment and for medicinal purposes [16]. Mercury occurs naturally in both liquid and vapor forms and can form compounds with various metals, particularly silver (Ag) and gold (Au). Since the early 19th century, Hg has also been widely used to produce amalgams with these metals. Over time, its applications have expanded to include Ag and Au extraction, the chlor-alkali industry, agriculture, and pharmaceutical products [17]. Organomercury compounds, including methylmercury (MeHg) and ethylmercury (EtHg), have also been used in certain vaccine formulations [18]. In the environment, Hg can undergo microbial methylation, resulting in the formation of MeHg, which can enter and bioaccumulate within food chains. Despite its long history of use and diverse applications, Hg is now recognized as a highly toxic and widespread environmental contaminant capable of adversely affecting multiple biological systems, even at relatively low concentrations [19,20].

Human exposure to heavy metals such as Pb, Cd, and Hg is widespread and occurs through multiple environmental and occupational sources, including agriculture, industrial processes, pharmaceuticals, pyrotechnic materials, and ammunition. Additional exposure may result from environmental contamination, urban pollution, and activities involving the use of ammunition, during which metal particles and residues are released into the surrounding environment [21]. Although the toxic effects of heavy metals on human health are well documented, the cellular and molecular mechanisms underlying their impact on the CNS, particularly those involving glial cell responses, remain incompletely understood.

Glial cells are among the first cellular components of the CNS to respond to environmental stressors and toxic insults. Despite their essential neuroprotective functions, they are highly susceptible to metal-induced toxicity. Both essential and non-essential metals can accumulate in the brain, promoting microglial activation and triggering neuroinflammatory responses. These processes may interact with other mechanisms of metal-induced neurotoxicity, including oxidative stress, mitochondrial dysfunction, and impaired synaptic regulation, ultimately contributing to neuronal dysfunction and degeneration [22].

Evidence from experimental rat studies further demonstrates the ability of heavy metals to accumulate in the brain. Brain concentrations of Pb, Cd, and Hg were higher in exposed rats than in control animals, with the magnitude of accumulation depending on the exposure concentration and age (Table 1). Pb levels were 6–30-fold higher in pups and 5–1200-fold higher in adults [23,24,25,26,27,28]; Cd levels were approximately 2-fold higher in pups and 5-fold higher in adults [28,29,30]; whereas Hg levels were 2–910-fold higher in pups and 71–5000-fold higher in adults [31,32,33,34]. These findings demonstrate substantial accumulation of heavy metals in both the developing and adult rat brain and further support the use of experimental rat models for investigating metal-induced neurotoxicity and glial responses.

Although the effects of trace elements and heavy metals on the nervous system have been extensively investigated, a comprehensive analysis integrating the available evidence on the use of experimental animal models to study glial responses to metal exposure remains lacking. In particular, rat model represents one of the most widely used and valuable experimental systems for studying glial cell biology because of its anatomical, physiological, and molecular similarities to the mammalian CNS, as well as the availability of well-established methods for assessing glial activation, neuroinflammation, and neurotoxic mechanisms (Figure 1). Therefore, this systematic review aims to analyze the available evidence on the use of rat models in studies of glial cells, with particular emphasis on their role in elucidating the cellular mechanisms underlying heavy metal-induced neurotoxicity.

## 2. Results and Discussion

### 2.1. Cadmium

Based on an analysis of scientific publications indexed in the PubMed database, research interest in the keywords “glial cells,” “cadmium,” and “rats” varied over time. The publications identified in the analysis were published between 1965 and 2024. The highest number of publications was recorded in 2005 (*n* = 7), followed by 1996 and 2008 (*n* = 6 each). In total, 88 articles were identified. After abstract-based screening and exclusion of articles that did not meet the predefined eligibility criteria, 77 publications were eligible for the next stage. Following full-text assessment for eligibility and compliance with the inclusion criteria, five scientific publications were ultimately included in the review (Figure 2).

Detailed information regarding the experimental conditions and methodologies used in the analyzed studies is presented in Table 2. The selected studies addressed several aspects of Cd-induced neurotoxicity, including oxidative stress, cellular antioxidant responses, BBB alterations, changes in glial cell populations, and myelin damage.

#### 2.1.1. Molecular Basis of Cadmium Toxicity

Cadmium occurs predominantly as Cd^2+^ under physiological conditions and, unlike redox-active metals such as iron or copper, does not readily undergo redox cycling. Therefore, Cd-induced oxidative stress is considered to arise primarily from disruption of cellular redox homeostasis rather than from direct redox reactions of Cd^2+^. At the molecular level, Cd^2+^ has a high affinity for sulfhydryl-containing molecules and proteins and can interact with glutathione and protein thiol groups, thereby impairing thiol-dependent antioxidant defenses. Cd can also interfere with the homeostasis of essential metal ions, particularly Zn^2+^ and Ca^2+^, by competing for or displacing these ions from protein-binding sites. Such interactions may alter the activity of enzymes, transcription factors, ion-dependent signaling pathways, and proteins involved in cellular stress responses.

These primary molecular disturbances can affect mitochondrial function and antioxidant capacity, resulting in secondary accumulation of reactive oxygen species (ROS), lipid peroxidation, and activation of stress-responsive pathways. Cd-induced oxidative stress may subsequently affect the endoplasmic reticulum, mitochondrial integrity, autophagic processes, and apoptotic signaling [38,39]. Thus, ROS accumulation following Cd exposure should be interpreted primarily as a downstream consequence and amplifier of disrupted thiol metabolism, metal homeostasis, and mitochondrial function rather than as a result of direct redox cycling by Cd^2+^.

An important cellular defense mechanism against Cd is the induction of metallothioneins (MTs). These cysteine-rich proteins bind Cd^2+^ and other metal ions and may reduce the availability of intracellular Cd, thereby limiting its interaction with critical cellular targets. Consequently, MT induction can be interpreted as an adaptive response to Cd exposure. However, under prolonged or excessive exposure, the capacity of this sequestration system may become insufficient, allowing Cd to remain biologically active and contributing to persistent cellular stress and toxicity. Thus, the relationship between Cd exposure and MT expression may reflect a dynamic balance between cellular sequestration and the toxic burden imposed by persistent Cd accumulation.

#### 2.1.2. Cadmium Accumulation, Metallothioneins, and the Blood–Brain Barrier

Ibiwoye et al. [40] investigated the effects of Cd exposure on the BBB and astrocytes in male Sprague-Dawley rats using indirect immunofluorescence with antibodies against endothelial barrier antigen (EBA) and glial fibrillary acidic protein (GFAP). Following intraperitoneal administration of CdCl_2_, decreased EBA immunoreactivity and a reduction in the number of EBA-positive microvessels were observed in the forebrain, cerebellum, and midbrain. Weak GFAP immunoreactivity was also observed in astrocytes in both white and gray matter. These findings suggest that Cd exposure can affect both the vascular and glial components of the neurovascular unit and may compromise BBB-associated functions [40].

The role of MTs in Cd handling has also been investigated in experimental studies involving male and pregnant female Wistar rats and pregnant ddY mice. Cd exposure induced MT expression, including in rat glial cells. MT levels were higher in young and adult rats than in mice and were higher in young rats than in older animals. The authors emphasized that prolonged Cd exposure was associated with MT expression in glial cells and with changes in choroid plexus epithelial and stem cell proliferation. These findings support the interpretation that MT induction represents an adaptive cellular response facilitating Cd sequestration and limiting its interaction with other intracellular targets.

From a mechanistic perspective, glial cells may therefore have a dual role in Cd toxicity. On the one hand, Cd accumulation within glial cells may increase their exposure to the metal and its molecular effects. On the other hand, glial cells may contribute to Cd sequestration through MT induction, potentially limiting the availability of Cd for interaction with other cellular components. When exposure is prolonged or excessive, however, these protective mechanisms may become insufficient, allowing sustained disruption of redox and metal homeostasis. BBB and choroid plexus may therefore represent important interfaces between circulating Cd and the CNS, influencing both Cd distribution and the subsequent cellular response.

#### 2.1.3. Oxidative Stress and Astrocyte Dysfunction

Unno et al. [35] investigated the effects of CdCl_2_ intoxication on sleep disturbances and locomotor activity in adult male Sprague-Dawley rats. Cd exposure increased the duration of non-REM sleep and reduced nocturnal locomotor activity during the active phase. At the molecular level, an increased proportion of oxidized glutathione (GSSG) was detected in the brain and astrocytes, indicating a shift toward a more oxidizing intracellular environment. The authors concluded that the observed changes in sleep behavior were associated with Cd-induced oxidative stress and suggested that increased non-REM sleep during the active phase may represent a functional indicator of acute Cd exposure [35].

The increase in GSSG is mechanistically consistent with the proposed effects of Cd on thiol-dependent antioxidant defenses. Because Cd^2+^ does not readily participate in direct redox cycling, the observed oxidative imbalance is more plausibly interpreted as a consequence of impaired glutathione homeostasis and other cellular defense mechanisms. Persistent disruption of the glutathione (GSH)/GSSG balance may subsequently affect mitochondrial function, protein homeostasis, and cellular signaling, thereby increasing the susceptibility of astrocytes and neighboring neural cells to secondary oxidative damage.

#### 2.1.4. Effects on Microglia, Oligodendrocytes, and Neuroendocrine Regulation

Saedi et al. [36] analyzed the effects of CdCl_2_ exposure on neuroanatomical changes in the hypothalamus of prepubertal female Sprague-Dawley rats. Cd exposure decreased the number of neurons and oligodendrocytes while increasing the number of microglial cells in the arcuate nucleus (ARC) and dorsomedial hypothalamus (DMH). These cellular changes were accompanied by decreased GnRH mRNA levels, increased RFRP-3 mRNA levels, and reduced concentrations of the gonadotropins luteinizing hormone (LH) and follicle-stimulating hormone (FSH). These findings indicate that Cd exposure during a sensitive developmental period can alter neuronal and glial cell populations and interfere with regulatory mechanisms of the hypothalamic–pituitary–gonadal (HPG) axis [36].

The increase in microglial cell number may indicate a reactive response to Cd-induced cellular stress or secondary tissue injury. However, the available evidence does not establish whether Cd directly activates specific molecular pathways in microglia or whether the observed microglial response occurs secondarily to neuronal, vascular, or metabolic disturbances. Similarly, the reduction in oligodendrocytes suggests that cells involved in myelin maintenance may be vulnerable to Cd-induced disruption of cellular homeostasis. These findings therefore support an effect of Cd on glial populations but do not, by themselves, establish a specific direct molecular target in these cells.

#### 2.1.5. Schwann Cell Injury and Disruption of Myelin Integrity

Sato et al. [37] observed that long-term administration of relatively low concentrations of CdCl_2_ dissolved in drinking water induced peripheral neuropathy in rats without hemorrhagic changes. In exposed animals, myelin degeneration and demyelination beginning at the node of Ranvier were observed, together with autophagy of Schwann cells accompanied by myelin debris, an increased number of ribosomes, and the formation of axoplasmic glycogenosomes. These findings suggest that chronic Cd exposure can disrupt Schwann cell homeostasis and myelin maintenance, potentially contributing to peripheral nerve dysfunction [37].

The observed autophagic changes may represent an adaptive response to Cd-induced cellular stress, aimed at removing damaged organelles and cellular components. However, persistent or excessive activation of autophagy may become dysregulated and contribute to cellular injury. Thus, the findings of Sato et al. [37] are compatible with a model in which prolonged Cd exposure induces cellular stress that initially activates protective mechanisms but ultimately compromises Schwann cell function and myelin integrity.

#### 2.1.6. Integrated Mechanistic Interpretation

Taken together, the available rat studies suggest that Cd-induced glial toxicity is not mediated by a single molecular target but rather by interconnected disturbances in thiol metabolism, antioxidant defense, cellular metal homeostasis, mitochondrial function, and glial-specific support processes. A common mechanistic pattern can be identified across the available studies. Cd^2+^ can interact with thiol-containing molecules and proteins, interfere with essential metal homeostasis, and induce metallothionein expression. These effects can alter intracellular redox balance and mitochondrial function, resulting in secondary ROS accumulation and activation of downstream cellular stress pathways [38,39].

The increase in oxidized glutathione observed in the brain and astrocytes provides in vivo evidence of altered redox homeostasis [35], whereas MT induction may represent an adaptive response that limits intracellular Cd availability and facilitates its sequestration [40]. However, prolonged exposure may exceed the capacity of these protective mechanisms, resulting in persistent oxidative stress and impairment of cellular functions. The effects observed in astrocytes and at the BBB suggest that glial and vascular components may influence Cd distribution and CNS responses, while the increased number of microglia and reduced number of oligodendrocytes indicate differential responses among glial populations [36,40]. In peripheral nervous tissue, Schwann cell autophagic alterations and myelin degeneration further demonstrate that chronic Cd exposure can impair glial support and myelin maintenance [37].

Overall, the available evidence supports a mechanistic model in which Cd-induced neurotoxicity begins with intracellular Cd accumulation and interactions with critical thiol-containing molecules, accompanied by disruption of essential metal homeostasis and induction of metallothioneins. These primary molecular effects may compromise antioxidant and mitochondrial function and subsequently activate or dysregulate downstream processes, including oxidative stress, autophagy, inflammatory responses, and cell death. Importantly, several molecular and cellular changes observed in the reviewed studies, including increased GSSG, MT induction, altered microglial and oligodendrocyte populations, and autophagic responses, should not necessarily be regarded as direct molecular targets of Cd. Some may represent adaptive, compensatory, or secondary responses to the initial cellular disturbance. Distinguishing direct molecular interactions from downstream responses is therefore essential for understanding the mechanisms underlying Cd-induced glial and neural toxicity.

### 2.2. Lead

Based on the analysis of scientific publications indexed in PubMed, 1501 articles addressing the keywords “glial cells,” “lead,” and “rats” and published between 1965 and 2024 were identified. Research activity increased over time, with the highest number of publications recorded between 2011 and 2017 (*n* = 500). Of these publications, 558 were available in full text and 555 were published in English (Figure 1). Following full-text assessment according to the predefined eligibility criteria, nine studies were included in the present review because they provided relevant experimental evidence regarding the effects of Pb exposure on glial cells and the central nervous system. Detailed characteristics of the included studies are presented in Table 3.

#### 2.2.1. Cellular Accumulation and Molecular Determinants of Pb Toxicity

Lead is predominantly present in biological systems as Pb(II), mainly as the Pb^2+^ ion, and can interact with cellular proteins, membranes, and signaling systems because of its ability to interfere with the binding of physiologically essential metal ions and to interact with metal- and thiol-containing biomolecules [47].

The biological effects of Pb therefore depend not only on the external dose but also on its absorption, transport in the circulation, tissue distribution, cellular uptake, intracellular sequestration, and duration of exposure. Within the nervous system, astrocytes appear to represent an important cellular compartment for Pb accumulation and sequestration [48]. Although this accumulation may initially limit the availability of Pb to other neural cells, persistent exposure may impair astrocytic homeostatic functions and contribute to secondary neuronal and glial injury [27].

An important feature of Pb(II) neurotoxicity is therefore the absence of a single molecular target. The evidence reviewed here indicates that Pb affects several interconnected cellular processes, including redox homeostasis, inflammatory signaling, autophagy, purinergic signaling, synaptic function, and myelin maintenance. These pathways may interact with each other, such that an initial disturbance in cellular signaling or metal homeostasis can result in oxidative stress and inflammatory activation, followed by impairment of cellular survival mechanisms and neural function [47].

#### 2.2.2. Oxidative Stress, Inflammation, and Autophagy

Oxidative stress represents one of the most consistent molecular responses to Pb(II) exposure observed across the included studies. Pb(II)-induced oxidative imbalance may result from interference with antioxidant systems and metal-dependent cellular processes, leading to increased oxidative damage and impaired cellular defense mechanisms [27,48]. In astrocytes, Huang et al. [41] reported increased levels of TNF-α, IL-1β, MDA, and MPO following Pb(II) acetate exposure, together with increased GFAP, LC3II, and Beclin-1 and decreased p62. The inhibitory effect of 3-methyladenine (3-MA) further supported the involvement of autophagy in the observed response. These findings suggest that Pb(II) exposure is associated with activation of autophagic pathways in astrocytes and that this response occurs together with inflammatory and oxidative changes. Inhibition of the Akt/mTOR pathway provides a potential molecular link between Pb(II) exposure, altered cellular signaling, and autophagy [41].

Importantly, the relationship between oxidative stress, inflammation, and autophagy should not be considered as a series of independent effects. Oxidative stress can activate stress-responsive signaling pathways, whereas inflammatory mediators can further impair cellular homeostasis. Conversely, dysregulated autophagy may influence the removal of damaged organelles and thereby modify the cellular response to oxidative injury. The findings of Huang et al. [41] therefore provide evidence for an interconnected network rather than a single linear pathway.

Further evidence for direct cellular injury was provided by Tang et al. [42], who compared the effects of lead acetate on astrocytes and Schwann cells. Pb(II) acetate exposure caused cellular vacuolization and ultrastructural alterations, including increased lysosomal content and endoplasmic reticulum expansion in astrocytes and mitochondrial enlargement, vacuolization, and myeloid bodies in Schwann cells. Increased lactate dehydrogenase release at higher Pb concentrations indicated loss of cellular integrity. The greater sensitivity of Schwann cells observed in this study suggests that glial cell type and intracellular handling of Pb may influence susceptibility to toxicity [42].

#### 2.2.3. Neuroinflammatory Signaling and Neuron–Glia Interactions

Evidence from in vivo studies indicates that Pb(II) exposure can induce persistent glial activation accompanied by inflammatory signaling. Strużyńska et al. [23] demonstrated increased GFAP and S-100β expression together with elevated IL-1β, TNF-α, IL-6, and CX3CL1 in the brains of immature rats exposed to Pb(II) acetate. At the same time, reductions in synapsin I and synaptophysin were observed in the hippocampus. These findings indicate that Pb-induced glial activation may be associated with impaired neuron–glia communication and synaptic dysfunction. CX3CL1 is particularly relevant in this context because the CX3CL1/CX3CR1 signaling system contributes to communication between neurons and microglia, suggesting a possible link between Pb(II) exposure, glial activation, neuroinflammation, and neuronal dysfunction [23,49].

The involvement of inflammatory transcriptional signaling was further demonstrated by Wei et al. [43], who observed Pb-induced COX-2 expression in glial and neuronal cell models. Pb(II) exposure was associated with activation of the NFAT pathway, supporting the involvement of the NFAT3/COX-2 axis in Pb-induced inflammatory responses. However, these findings demonstrate involvement of COX-2 and NFAT signaling rather than proving that Pb directly binds to or targets these proteins. Thus, COX-2 and NFAT should be considered molecular pathways implicated in Pb(II) toxicity rather than established direct molecular targets.

#### 2.2.4. Purinergic Signaling and Peripheral Glial Responses

The effects of Pb are not restricted to astrocytes and CNS inflammation. Studies examining satellite glial cells in sympathetic ganglia indicate that Pb(II) exposure may also alter purinergic signaling and autonomic regulation. Zhu et al. [44] reported increased P2X7 receptor expression in satellite glial cells together with enhanced sympathetic activity, increased blood pressure and heart rate, and reduced heart rate variability following chronic Pb(II) acetate exposure. In a related study, increased P2X4 receptor expression, IL-1β, connexin 43, and phosphorylation of p38 MAPK were observed in stellate ganglia following chronic Pb(II) exposure. These findings suggest that altered purinergic signaling in peripheral glial cells may contribute to neuroinflammatory responses and enhanced sympathetic excitability. Importantly, increased P2X4/P2X7 expression should be interpreted as evidence of pathway involvement rather than proof of direct Pb–receptor interaction.

#### 2.2.5. Effects on Myelin and Neuronal Development

Several studies suggest that Pb-induced glial dysfunction may ultimately impair myelin integrity and neuron–glia interactions. Zawia and Harry [24] reported altered expression of GAP-43, MBP, and GFAP during postnatal development following 0.2% Pb(II) acetate exposure, indicating changes in neuronal growth and glial and myelin-associated processes. Latronico et al. [45] further demonstrated increased susceptibility of myelin proteins to ROS-mediated damage and increased MBP-degrading activity in Pb(II)-exposed animals. These findings provide a potential mechanistic link between Pb-induced oxidative stress and impaired myelin maintenance. Because oligodendrocytes and Schwann cells are responsible for maintaining myelin, disruption of redox homeostasis and increased degradation of myelin-associated proteins may contribute to the morphological and functional abnormalities observed after developmental or chronic Pb(II) exposure.

#### 2.2.6. Toxicokinetic and Bioaccumulation-Related Considerations

The interpretation of Pb-induced glial toxicity should also take into account the relationship between exposure, circulation, and tissue accumulation. Rojas-Castañeda et al. [46] detected Pb(II) acetate in blood, hypothalamus, and hippocampus following chronic exposure and observed morphological alterations and reduced cellular density in the suprachiasmatic nucleus. These findings support the relevance of tissue distribution and prolonged Pb retention to CNS effects. However, total tissue Pb concentration does not necessarily reflect the concentration of biologically reactive Pb at a specific molecular target. Cellular sequestration, protein binding, subcellular distribution, and exposure duration may determine whether Pb produces adaptive responses or progresses to cellular dysfunction.

#### 2.2.7. Integrated Mechanistic Interpretation

Taken together, the studies included in this review indicate that Pb-induced glial toxicity is best understood as a network of interconnected molecular and cellular disturbances rather than as the consequence of a single molecular target. A recurring pattern across the available studies consists of altered cellular Pb handling followed by disruption of redox homeostasis and intracellular signaling, activation of inflammatory pathways, and changes in autophagy and purinergic signaling. These processes may subsequently impair neuron–glia communication, synaptic function, mitochondrial and membrane integrity, and myelin maintenance. The available evidence therefore supports a mechanistic sequence in which Pb(II) exposure and cellular accumulation initiate or amplify intracellular stress responses, while oxidative and inflammatory signaling contribute to progressive glial dysfunction and neural injury.

Nevertheless, the strength of evidence differs among the proposed mechanisms. Increased cytokine expression, oxidative stress markers, autophagy-related proteins, P2X receptor expression, and myelin-associated changes are well documented in the included experimental models, whereas direct molecular interactions between Pb and individual signaling proteins or receptors remain less clearly established. Accordingly, the mechanisms discussed above should be distinguished between experimentally demonstrated cellular responses and molecular interactions that remain mechanistically proposed.

### 2.3. Mercury

Based on an analysis of scientific publications indexed in the PubMed database, the research interest in the keywords “glial cells,” “mercury,” and “rat” varied over time. The identified publications were published between 1970 and 2024, with the highest number of publications recorded in 1991 (*n* = 6), followed by 1996 and 2001 (*n* = 5 each). In total, 94 articles were identified. After abstract screening and exclusion of studies that did not meet the predefined eligibility criteria, 15 publications were selected for further assessment. Following full-text evaluation, five studies met the inclusion criteria and were included in the review (Figure 1). Detailed information regarding the experimental conditions and methodologies used in these studies is presented in Table 4.

#### 2.3.1. Mercury Speciation and Determinants of Cellular Toxicity

The neurotoxic effects of Hg depend not only on the dose and duration of exposure but also on its chemical form, absorption, transport, tissue distribution, cellular uptake, intracellular sequestration, and the susceptibility of individual neural cell types. Importantly, elemental mercury (Hg^0^), inorganic mercury (IHg; predominantly Hg^2+^ under physiological conditions), and organic mercury compounds such as MeHg differ substantially in their physicochemical properties, toxicokinetics, cellular transport, and molecular reactivity [56]. Consequently, findings obtained with one Hg species should not be directly generalized to other forms of mercury.

MeHg is an organic mercury compound that can be formed from inorganic mercury through microbial methylation in aquatic environments. Its accumulation in aquatic organisms and subsequent biomagnification in food webs represent important routes of exposure for humans and other organisms. The ability of MeHg to cross biological barriers and enter cells contributes to its pronounced neurotoxicity, particularly during brain development [57]. By contrast, inorganic Hg species exhibit different distribution and cellular uptake characteristics and may show greater retention in particular tissues or cellular compartments. Therefore, the biological effects of Hg should be interpreted in the context of Hg speciation rather than total Hg concentration alone.

Although tissue Hg concentration is frequently used as an indicator of exposure, total Hg burden does not necessarily predict the severity or cellular localization of neurotoxicity. Experimental evidence suggests that Hg can accumulate preferentially in glial cells and may subsequently be detected in neurons, indicating that cellular uptake, intracellular binding, sequestration, and efflux mechanisms may contribute to cell-type-specific susceptibility [58]. Astrocytes are particularly relevant targets of MeHg toxicity because they participate in glutamate and glutamine metabolism, antioxidant defense, and maintenance of neuronal homeostasis. Thus, disruption of astrocytic functions may contribute indirectly to neuronal injury even when the initial molecular effects occur within glial cells.

At the molecular level, Hg species exhibit a high affinity for thiol- and selenol-containing molecules. Interaction with GSH, protein thiols, and selenium-dependent proteins may impair antioxidant defenses and alter cellular redox homeostasis [59]. These effects can result in secondary ROS accumulation, lipid peroxidation, mitochondrial dysfunction, and activation of cell-death pathways [60]. Accordingly, oxidative stress observed after Hg exposure should be considered both a consequence of direct interactions between reactive Hg species and cellular macromolecules and a potential amplifier of downstream cellular injury [59].

#### 2.3.2. Methylmercury Uptake and Glial Cell Susceptibility

The studies included in the present review predominantly investigated MeHg, providing evidence that cellular uptake and intracellular handling contribute to differential susceptibility among neural cell types. Yoshida et al. [50] investigated the mechanisms underlying the differential susceptibility of sensory neurons and glial cells to MeHg by comparing mercury accumulation and the expression of transporters involved in MeHg uptake and efflux. Dorsal root ganglion (DRG) cells, anterior horn cells (AHCs), and Schwann cells obtained from 3–4-week-old Wistar rats were examined. The authors assessed the expression of L-type amino acid transporter 1 (LAT1), which may contribute to MeHg uptake, and multidrug resistance-associated protein 2 (MRP2), which is involved in the efflux of MeHg–glutathione conjugates. MeHg accumulated preferentially in DRG cells and was associated with reduced cell viability. These findings indicate that differences in transporter expression and intracellular mercury handling may contribute to cell-type-specific susceptibility to MeHg [50].

The importance of cellular uptake and thiol status was further demonstrated by Ni et al. [51], who compared the responses of astrocytes and microglia isolated from one-day-old Sprague-Dawley rats following MeHg exposure. Microglia exhibited greater mercury uptake, lower GSH levels, and increased ROS production than astrocytes, together with higher Nrf2 expression. These findings suggest that the greater susceptibility of microglia may be associated with more pronounced mercury accumulation and depletion of cellular thiol reserves. Increased Nrf2 expression may represent a compensatory response to oxidative stress rather than a direct toxic effect of MeHg. Collectively, these findings indicate that cellular mercury burden and the capacity to maintain thiol-dependent antioxidant defenses are important determinants of glial susceptibility [51].

#### 2.3.3. Oxidative Stress, Glutamate Homeostasis, and Mitochondrial Dysfunction

Oxidative stress and disruption of neurotransmitter homeostasis emerge as recurrent mechanisms of MeHg-induced neural injury. Yin et al. [52] examined the effects of MeHg on oxidative damage, mitochondrial membrane potential, glutamine uptake, and glutamate-related transport in cortical astrocytes obtained from Sprague-Dawley rats. Exposure to 5–10 μM MeHg for 1–6 h increased ROS generation and F2-isoprostanes, indicating enhanced lipid peroxidation, and disrupted membrane permeability and the glutamine–glutamate cycle. These findings suggest that MeHg-induced oxidative damage may be closely linked to impaired astrocytic metabolic and neurotransmitter-support functions.

The disruption of astrocytic glutamate homeostasis is particularly relevant because astrocytes are responsible for removing extracellular glutamate and maintaining the glutamate–glutamine cycle. Impairment of these processes may increase extracellular glutamate availability and thereby enhance neuronal excitotoxic vulnerability. Thus, MeHg-induced oxidative stress and mitochondrial dysfunction may contribute to neuronal injury not only through direct cellular damage but also through disruption of essential glial support functions [52].

Mercury-induced oxidative stress may also activate downstream apoptotic pathways. Experimental studies using inorganic mercury compounds, including HgCl_2_, have reported increased Bax and caspase-3 expression and decreased Bcl-2 levels, consistent with activation of mitochondrial apoptotic signaling [61]. Endoplasmic reticulum stress may further contribute to Hg-induced cellular injury and cell death [62]. Although these mechanisms are relevant to the broader toxicology of mercury, they should be interpreted cautiously in the context of the present review because the included rat studies predominantly investigated MeHg, whereas these particular mechanistic observations were obtained using inorganic mercury exposure.

#### 2.3.4. Blood–Brain Barrier and Vascular Responses

The blood–brain barrier represents another important determinant of the distribution and biological effects of Hg in the CNS. Takahashi et al. [53] investigated the effects of MeHg exposure on the BBB in rats. Although no major differences in mercury concentration were detected among the examined brain regions, MeHg exposure was associated with increased permeability of the BBB, demonstrated by IgG leakage into surrounding tissues and reduced endothelial antigen expression. Increased VEGF expression was observed in astrocytes and was associated with the vascular response to MeHg exposure. These findings suggest that astrocytes may participate in the response to MeHg-induced BBB dysfunction and that altered astrocyte–vascular interactions may contribute to CNS toxicity [53].

The relationship between mercury accumulation and BBB dysfunction is therefore potentially bidirectional. The chemical form of mercury influences its ability to reach the CNS, while Hg-induced changes in barrier integrity may subsequently alter the distribution of Hg and other circulating factors within the neural tissue. This mechanism may contribute to the persistence or amplification of neurotoxic responses following exposure.

#### 2.3.5. Developmental and Long-Term Effects of MeHg

The consequences of MeHg exposure may extend beyond acute cellular injury and may be particularly pronounced during neurodevelopment. Rustom and Reynolds [54] investigated the effects of developmental MeHg exposure in Sprague-Dawley rats. Following maternal exposure during gestation and lactation, offspring examined.

### 2.4. Integrated Comparison of Molecular Mechanisms of Hg, Pb, and Cd Toxicity

An important consideration when interpreting the available literature is the chemical form of the metals used experimentally. In the reviewed studies, Cd and Pb were predominantly administered as soluble salts, particularly cadmium chloride and lead acetate, and their effects should therefore be interpreted mainly in the context of ionic Cd^2+^ and Pb^2+^. Mercury requires greater attention to chemical speciation because elemental mercury (Hg^0^), inorganic mercury (Hg^2+^, IHg) and organic forms such as MeHg differ substantially in absorption, distribution, cellular uptake, binding, and molecular reactivity. Findings obtained with MeHg should therefore not be directly generalized to other mercury species.

The molecular effects of Hg, Pb, and Cd also depend on toxicokinetic processes, including absorption, systemic distribution, transport to the CNS, blood–brain barrier passage, cellular uptake, sequestration, and elimination [63]. Glutathione and metallothioneins are important determinants of intracellular metal availability and may initially provide protection by limiting the fraction of reactive metal. However, prolonged exposure can exceed these buffering mechanisms. Consequently, total tissue metal concentration does not necessarily reflect the amount of biologically reactive metal at molecular targets, and differences in cellular handling may contribute to the distinct susceptibilities of glial cell types.

Despite differences in speciation and cellular handling, several common mechanisms emerge. All three metals disrupt redox homeostasis, although through different molecular processes. Cd^2+^ promotes oxidative stress mainly through impairment of thiol-dependent antioxidant defenses, essential metal homeostasis, and mitochondrial function. Pb can interact with protein thiols and affect inflammatory, purinergic, and autophagy-related signaling. Hg species, particularly MeHg, strongly interact with thiol- and selenol-containing molecules and can disrupt glutamate homeostasis, mitochondrial function, and antioxidant defenses. Thus, oxidative stress is a common downstream feature but does not indicate an identical mechanism of toxicity.

Glial responses may initially be adaptive, involving metallothionein induction, Nrf2 signaling, glutathione utilization, and autophagy. With prolonged or intense exposure, these mechanisms may become insufficient, leading to persistent oxidative stress, inflammation, mitochondrial dysfunction, impaired neurotransmitter homeostasis, blood–brain barrier disruption, demyelination, and cell death. This may partly explain differences between acute and chronic exposure studies.

Overall, the evidence does not support a single molecular target common to Cd, Pb, and Hg (Table 5). Toxicity appears to result from interactions with chemically susceptible cellular components, followed by interconnected stress and signaling responses. Direct molecular effects are most plausibly related to binding to thiol- or selenol-containing molecules, disruption of essential metal-dependent proteins, and, for some Hg species, interference with transport processes. In contrast, changes in ROS, cytokines, GFAP, Nrf2, autophagy, or apoptotic markers should generally be regarded as downstream or adaptive responses unless direct molecular interactions have been demonstrated.

Overall, neurotoxicity induced by Hg, Pb, and Cd reflects the interplay between chemical speciation, toxicokinetics, cellular accumulation, molecular reactivity, and cell-specific defense mechanisms. Although oxidative stress, inflammation, mitochondrial dysfunction, altered autophagy, and impaired glial functions are recurrent findings, their initiating mechanisms and relative contributions differ between metals and exposure models.

## 3. Methods

Scientific publications from the fields of medicine and biological sciences were analyzed using the PubMed database. The review was conducted between 26 and 30 November 2024. The majority of the publications included in the analysis were published between 1965 and 2024. The following keywords were used: glial cells, cadmium, lead, mercury, and rats. The inclusion criteria comprised publications in peer-reviewed journals, including both review articles and original research articles published in English. The exclusion criteria included case reports, publications in languages other than English, abstracts, and conference materials.

Small rodents can be exposed to specific neurotoxic agents, enabling comprehensive investigations ranging from molecular mechanisms to complex behavioral responses [72]. Norway rats (*Rattus norvegicus*) offer several advantages as experimental models, including similarities in brain anatomy to humans, a relatively short lifespan, rapid reproduction, small body size, cost-effectiveness, and ease of housing and maintenance. In addition, rats are well suited for behavioral testing and toxicological studies, making them valuable experimental models in biomedical research [73]. Rats were selected for this study because their glial cells and neurobiological characteristics have been extensively characterized, making this species a well-established and suitable model for neurotoxicological research [74].

The literature analysis was conducted in accordance with PRISMA guidelines [75]. A predefined protocol was registered with the International Prospective Register of Systematic Reviews (PROSPERO; CRD420261434949).

## 4. Conclusions

Brain accumulation of Hg, in particular, should be considered not merely a marker of exposure but an important determinant of its neurotoxic effects. Although methylmercury and inorganic Hg differ in their toxicokinetic properties and cellular targets, both may converge on mechanisms involving oxidative stress, impaired antioxidant defenses, mitochondrial dysfunction, and altered inflammatory signaling. Similarly, Cd, Pb, and Hg differ in their physicochemical properties and molecular targets, but their effects may converge on several fundamental cellular processes, supporting the concept of glial dysfunction as a shared component of their neurotoxicity.

In summary, this study aimed to investigate the effects of cadmium, lead, and mercury on the structure and function of glial cells. The findings of the analyzed studies indicate that these three heavy metals induce alterations in glial cells at morphological, physiological, and developmental levels. Given the essential role of glial cells in maintaining brain homeostasis and supporting neuronal function, such alterations may have profound consequences for the proper functioning of the central nervous system. The neurotoxic effects of these metals are associated with oxidative stress, disruption of cellular homeostasis, morphological changes, and, in severe cases, the induction of apoptosis (Figure 2). Importantly, the effects of lead, cadmium, and mercury extend beyond glial cells and the central nervous system, affecting a range of physiological processes throughout the organism. Even relatively low levels of exposure to these toxic elements may interfere with processes such as circadian rhythm regulation, highlighting the broad systemic impact of heavy-metal toxicity.

The use of well-established animal models has contributed substantially to understanding these mechanisms. Sprague–Dawley and Wistar rats are widely used in neuroscience and neurotoxicological research because of their well-characterized brain structure, reproducible physiological and behavioral responses, and established sensitivity to environmental neurotoxicants, including heavy metals. These characteristics make them valuable models for investigating the cellular and molecular mechanisms underlying heavy-metal-induced neurotoxicity and for evaluating the consequences of exposure at the organismal level.

Overall, the studies discussed in this work provide important insights into the mechanisms underlying the toxic effects of cadmium, lead, and mercury on glial cells and the nervous system. They also contribute to a better understanding of the potential links between environmental exposure to these elements and the etiology and progression of neurological disorders. Elucidating these mechanisms represents an important step toward developing more effective strategies for preventing and mitigating the adverse effects of environmental pollutants on the nervous system.

## Figures and Tables

**Figure 1:**
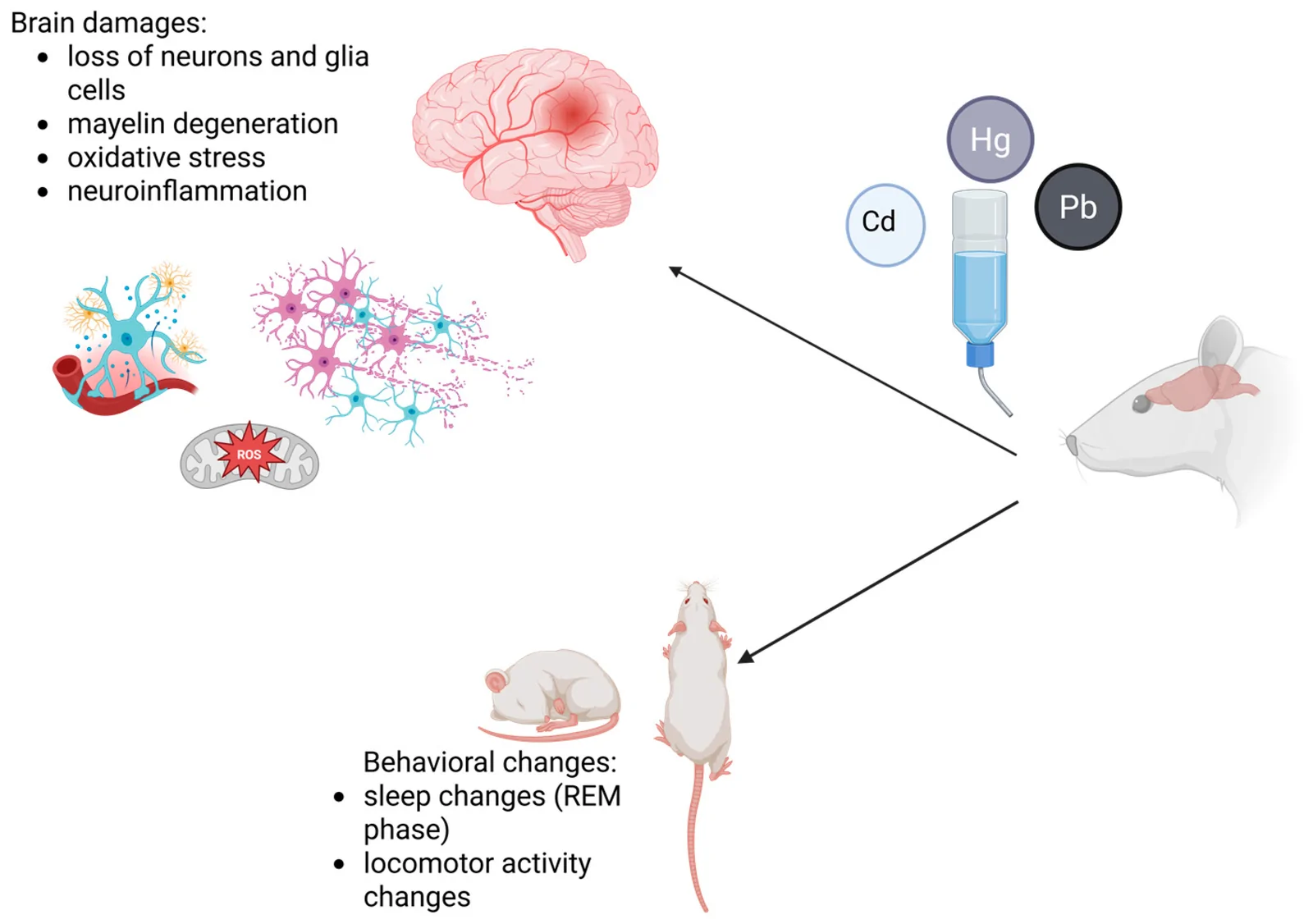
Toxic metals action in rat brain. Created in BioRender. Physiology, D. (2026) https://BioRender.com/wn110j9 (accessed on 7 August 2026).

**Figure 2:**
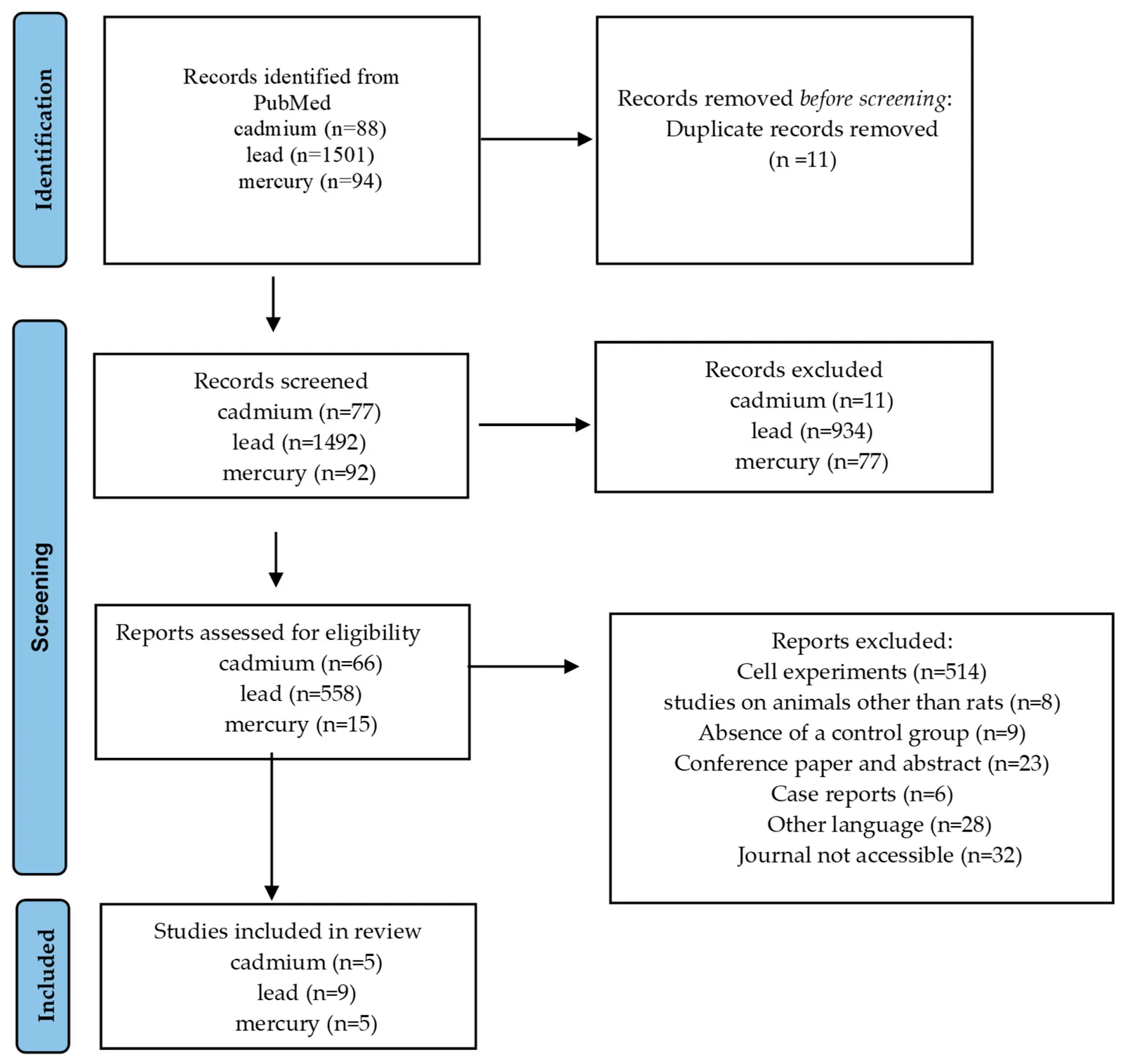
Diagram illustrating the process of searching and selecting scientific literature for the keywords “glial cells,” “cadmium,” “lead,” “mercury,” and “rats” using the PubMed database.

**Table 1:** Brain concentrations of lead (Pb), cadmium (Cd) and mercury (Hg) in experimentally exposed rat pups and adults.

Strain	Developmental Stage	Exposed Group		Control Group	Citation
		Method of Administration and Dosage	Concentration in the Brain		
Pb					
Wistar	Pups	Daily intraperitoneal injection of Pb-acetate (15 mg/kg)	0.35 ± 0.09 µg/g	<0.02 µg/g	[23]
Long–Evans	Pups	0.2% Pb-acetate to the deionized boiled drinking water of the dam and continued until PND 20	0.25 ± 0.07 mg/g	0.08 mg/g	[24]
Sprague–Dawley	Pups	0.2% Pb-acetate in the drinking water of the dam from gestational day 15 to postnatal day 21	0.686 ± 0.147 µg/g	0.113 ± 0.093 µg/g	[25]
Wistar	adult male	0.5% (*v*/*v*) Pb- acetate in drinking water was administrated chronically over a 3-month period	0.0068 ± 0.0013 µg/g	0.0018 ± 0.0008 µg/g	[26]
Wistar	Adult male	Pb-acetate (25 mg/kg b.w.), i.p. injection, for 3 consecutive days.	2.4 ± 0.3 µg/g b.w	<0.0019 µg/g w.w.	[27]
Wistar	Adult male	gavage of Pb solution (2 mg/kg) every other day for 4 weeks	0.091 ± 0.04 µg/g	0.019 ± 0.005 µg/g	[28]
Cd					
Wistar	male	Cd solution (2.5 mg/kg, p.o.) administered every other day for 4 weeks	0.74 ± 0.063 µg/g	0.14 ± 0.029 µg/g	[28]
Sprague-Dawley	Offspring	5 ppm Cd in the drinking (5 mg/L)	<0.004 mg/kg w.w.	<0.004 mg/kg w.w.	[29]
Drukery	Pups	Cadmium acetate (50 ppm) administered via drinking water daily from gestational day 0 until delivery	0.32 ± 0.05 µg/g	0.15 ± 0.01 µg/g	[30]
Hg					
Sprague-Dawley	Offspring	MeHgCl in diet: 3.9 ± 1.2 mg Hg/kg diet; maternal exposure (14 weeks pre-mating + gestation/lactation); offspring exposure via milk and diet until PND50	1.45 ± 0.06 mg/kg w.w.	0.035 ± 0.003 mg/kg w. w.	[31]
Wistar	Pups	MeHgCl (1 mg/kg/day), orally, 10 days	2.34 ± 0.08 µg/g w.w.	1.58 ± 0.04 µg/g w.w.	[32]
Long Evans	Pups	0.5 ppm Hg (as MeHgCl) in drinking water	0.45 (M) 0.52 (F)	<0.01	[33]
		6 ppm Hg (as MeHgCl) in drinking water	0.30 (M) 9.1 (F)		
Brown Norwegian	Male and female	Hg vapor (~1 mg/m^3^): 24 h/day	5.03 ± 0.73 µg/g	0.01 ± 0.005 µg/g	[34]
		Hg vapor (~1 mg/m^3^): 6 h/day, 3 days/week	0.71 ± 0.1 µg/g		

w.w., wet weight; i.p., intraperitoneal; PND, postnatal days.

**Table 2:** Rat studies on cadmium chloride (CdCl_2_) exposure.

Model	Glial Cell Type	Dose	Exposure Time	Experimental Approach	Molecular Effects	Functional Outcomes	Citation
Sprague–Dawley rats, adult male	astrocytes	1–100 ppm in drinking water	28 h (100 ppm)	oral exposure, sleep and locomotor activity analysis	↑ oxidized glutathione (GSSG) in brain and astrocytes → oxidative stress	↑ non-REM sleep duration, ↓ nocturnal locomotor activity	[35]
Sprague–Dawley rats, prepubertal female	microglia, oligodendrocytes	25 mg/kg in oral gavage	not specified	oral administration, neuroanatomical and hormonal analysis	↓ GnRH mRNA, ↑ RFRP-3 mRNA	↓ neurons and oligodendrocytes, ↑ microglia (ARC, DMH), ↓ LH and FSH levels	[36]
Wistar rats of 5–6 weeks	Schwann cells	in drinking water: 10 ppm (9 months) → 20 ppm (3 months) → 30 ppm (2 months) → 40 ppm (until the end of the experiment)		oral exposure, histopathological analysis	↑ ribosomes in Schwann cells, autophagy	Demyelination (starting at nodes of Ranvier), myelin degeneration, peripheral neuropathy	[37]

↑ increase; ↓ decrease; → subsequent effect.

**Table 3:** Rat studies on lead acetate (Pb(CH,COO)_2_) exposure.

Model	Glial Cell Type	Pb Exposure Conditions	Exposure Time of Pb	Experimental Approach	Molecular Effects	Functional Outcomes	Citation
Sprague–Dawley rats + in vitro (primary astrocytes)	Astrocytes	300 ppm in drinking water	30 days	Oral exposure, immunohistochemistry, Western blot	↑ TNF-α, ↑ IL-1β, ↑ MDA, ↑ MPO, ↑ GFAP, ↑ LC3II, ↑ Beclin-1, ↓ p62; inhibition of Akt/mTOR pathway	Autophagy activation, ↑ neuroinflammation, ↑ oxidative stress	[41]
Sprague-Dawley rats	Astrocytes	0–100 μg/mL	4 days	Scanning and transmission electron microscopy, LDH release Assay	↑ lysosomes, ↑ ER, membrane changes at 10–100 μg/mL	Cytotoxicity, morphological changes, vacuolization, increased membrane damage	[42]
	Schwann cells	0–10 μg/mL	24 h		↑ mitochondria, vacuolization, myeloid-like cells at 1–10 μg/mL; ↑ LDH release in both cell types; no significant change in GSH or lipid peroxidation in astrocytes		
Wistar rats, pups	Astrocytes (GFAP, S-100β),	injected daily (15 mg/kg)	2 weeks	injection, biochemical and protein expression analysis	↑ GFAP, ↑ S-100β, ↑ IL-1β, ↑ TNF-α, ↑ IL-6, ↑ CX3CL1	Glial activation, neuroinflammation, ↓ synapsin I and synaptophysin (hippocampal damage)	[23]
	Microglia (indirect)						
in vitro (C6 glioma cells, BV2 microglia, others)	Astrocyte-like cells, Microglia	Not specified	Not specified	Cell culture, gene/protein expression analysis	↑ COX-2 expression, activation of NFAT pathway, ↑ oxidative stress and inflammation	Induction of neuroinflammatory response (NFAT3/COX-2 axis)	[43]
Sprague–Dawl ey rats	Satellite glial cells (SCG)	0.5–2 g/L in drinking water	10 days + long-term follow-up	Oral exposure, immunofluorescence, electrophysiology	↑ P2X7 receptor expression (protein and mRNA)	↑ sympathetic activity, hypertension, ↓ heart rate variability	[44]
Long–Evans rats	Astrocytes, Oligodendrocytes	0.2% drinking water	Until PND 20 (analysis PND 3–50)	Oral exposure, Northern blot	↑ GAP-43 mRNA, ↓ MBP, ↓ GFAP	Impaired neuron–glia interactions, developmental neurotoxicity	[24]
Wister rats, prenatal and postnatal exposure	Oligodendrocytes (myelin-related)	0.3 mg/mL in drinking water	during gestation and lactation; offspring exposure continued from PND2	Oral exposure, protein analysis	↑ susceptibility of myelin proteins to ROS, MBP degradation	Myelin damage, impaired myelin integrity	[45]
Wistar rats, pregnant rats and pups	Not specified	320 ppm in drinking water	Chronic (gestation and postnatal exposure until day 90)	Oral exposure, AAS, morphometric analysis	Not specified	↓ cell density and morphological alterations in SCN, circadian rhythm disruption	[46]

↑, increase; ↓, decrease.

**Table 4:** Rat studies investigating the effects of mercury species on glial and neural cells (dorsal root ganglion, DRG; anterior horn cells, AHCs).

Model	Glial Cell Type	Mercury Exposure Conditions	Exposure Time	Experimental Approach	Molecular Effects	Functional Outcomes	Citation
Wistar rats, 3–4 weeks	DRG cells, AHCs, Schwann Cells	MeHg, not reported	Not reported	LAT1 and MRP2 expression analysis, Hg accumulation	↑ LAT1, ↓ MRP2 in DRG, ↑ Hg Accumulation	Decreased cell viability in DRG	[50]
Sprague-Dawley rats, 1 day	Astrocytes, Microglia	MeHg, Not Reported	Not reported	ROS, GSH, Hg uptake, Nrf2 expression	Microglia: ↑ ROS, ↓ GSH, ↑ Hg uptake, ↑ Nrf2	Microglia more sensitive to MeHg than astrocytes	[51]
Sprague-Dawley rats	Cortical astrocytes	MeHg 5–10 μM	1–6 h	ROS, F2-isoprostanes, mitochondrial potential, glutamine cycle analysis	↑ ROS, ↑ F2-isoprostanes, disrupted glutamine-glutamate cycle	Cell dysfunction and death	[52]
Male Wistar rats, 6 weeks	Astrocytes	IHg, 20 ppm in drinking water	4 weeks	BBB analysis, Immunohistochemistry	↑ VEGF in astrocytes, inhibited endothelial antigen expression	BBB disruption, IgG leakage into tissue	[53]
Sprague-Dawley Rats, offspring	Astrocytes, microglia, neurons, endothelium	MeHg, 0.02 or 2.0 mg/kg Bw	Gestation and lactation	Immunohistochemistry for neuronal, glial, apoptotic, endothelial markers	↑ GAD67, ↑ RECA-1, ↓ lipofuscin	Structural and functional brain alterations in offspring	[54]
Wistar rats, male 90 days old	Cerebellar astrocytes	IHg, 0.375 mg/kg/day	45 days	Proteomic analysis, ACAP and LPO Measurements	Disrupted proteins related to synaptic signaling, metabolism, ↑ cytotoxicity	↑ apoptosis, cell degeneration, motor dysfunction	[55]

↑, increase; ↓, decrease.

**Table 5:** Integrated overview of chemical speciation, molecular targets, and major mechanisms of Hg, Pb, and Cd toxicity in glial cells.

Metal Species	Transport and Accumulation Mechanisms	Proposed Molecular Interactions and Mechanisms	Molecular Mechanisms and Signaling Pathways	Cellular Targets.	Downstream Outcome	References
Cd^2+^	transporters, BBB, MT sequestration	thiol groups, metal-binding proteins	redox signaling, mitochondrial stress, autophagy	astrocytes, microglia, oligodendrocytes, Schwann cells	oxidative stress, demyelination, cell death	[64,65]
Pb^2+^	transport resembling essential cations, tissue accumulation	protein thiols, metal-binding sites; signaling proteins	Akt/mTOR, NFAT/COX-2, MAPK, purinergic signaling	Astrocyte, microglia satellite glia	neuroinflammation, synaptic/myelin dysfunction	[66,67,68]
MeHg	amino-acid/thiol-related transport, BBB crossing, bioaccumulation	thiol/selenol groups, GSH-related systems	Nrf2/redox pathways, mitochondrial dysfunction, glutamate homeostasis	astrocytes, microglia, Schwann cells	oxidative stress, excitotoxicity, apoptosis	[60,69]
Hg^2+^	protein/thiol binding, tissue accumulation	thiol/selenol groups	mitochondrial/apoptotic pathways	glial	oxidative damage, apoptosis	[59,70,71]

## Data Availability

No new data were created or analyzed in this study. Data sharing is not applicable to this article.
